# Exploring the views of planners and public health practitioners on integrating health evidence into spatial planning in England: a mixed-methods study

**DOI:** 10.1093/pubmed/fdaa055

**Published:** 2020-05-19

**Authors:** Janet Ige-Elegbede, Paul Pilkington, Emma L Bird, Selena Gray, Jennifer S Mindell, Michael Chang, Aimee Stimpson, Dominic Gallagher, Carl Petrokofsky

**Affiliations:** 1 Centre for Public Health and Wellbeing, The University of the West of England, Stoke Gifford BS16 1QY, UK; 2 Department of Epidemiology and Public Health, UCL, London WC1E 6BT, UK; 3 Healthy Places, Priorities and Programmes Division, Health Improvement Directorate, Public Health England, London SE1 8UG, UK

**Keywords:** places, planning, public health

## Abstract

**Background:**

This study explored barriers and facilitators to integrating health evidence into spatial planning at local authority levels and examined the awareness and use of the Public Health England ‘Spatial Planning for Health’ resource.

**Methods:**

A sequential exploratory mixed-methods design utilized in-depth semi-structured interviews followed by an online survey of public health, planning and other built environment professionals in England.

**Results:**

Views from 19 individuals and 162 survey responses revealed high awareness and use of the Spatial Planning for Health resource, although public health professionals reported greater awareness and use than other professionals. Key barriers to evidence implementation included differences in interpretation and the use of ‘evidence’ between public health and planning professionals, lack of practical evidence to apply locally and lack of resource and staff capacity in local authorities. Key facilitators included integrating health into the design of local plans, articulating wider benefits to multiple stakeholders and simplifying presenting evidence (regarding language and accessibility).

**Conclusion:**

The Spatial Planning for Health resource is a useful resource at local authority level. Further work is needed to maximize its use by built environment professionals. Public health teams need support, capacity and skills to ensure that local health and well-being priorities are integrated into local planning documents and decisions.

## Introduction

It is widely recognized that the built environment can positively impact on population health and well-being.^1^^,^^2^ Built environment and public health professionals share a historically important role in facilitating the design of healthy spaces.^3^^,^^4^ However, despite the long and well-known history between planning and health in the UK, the two disciplines are, at present, not sufficiently integrated at local levels. There have been repeated calls for better synergy between planning and public health teams to enable the delivery of healthy places.^3^^,^^5–7^

The England National Planning Policy Framework (NPPF) recognizes the unique role of spatial planning in improving community health and well-being and calls for stronger partnerships between planning authorities and public health specialists in assessing the health needs of a community and addressing health inequalities.^8^ Health and well-being considerations should begin with the planning process and should not be an afterthought.^9^ One of the recent developments that have led to improvements in synergizing public health and planning in the UK is the relocation of public health teams from the National Health Service (NHS) to local authorities to promote close working with planning and other teams. ^10^^,^^11^ Despite this, effective collaborations between public health and planning teams remain threatened by not only capacity and resource issues but by other issues such as cultural differences between disciplines and differences in the way evidence is collected, used and interpreted to influence the different processes that are required for setting health and well-being priorities in the planning process.^7^

Several evidence-informed resources such as Spatial Planning for Health,^12^ the Healthy Urban Development Unit Rapid Health Impact Assessment Tool (HUDU)^13^ and Putting Health Into Place^14^ were developed to facilitate effective dialogue between built environment and public health professionals. The ‘Spatial Planning for Health’ Evidence Resource developed by Public Health England (PHE) aimed to provide public health professionals and planners in local communities with evidence-informed principles for designing healthy places. The Resource is an evidenced-based resource that examined the links between the built and natural environment and health in five areas (housing, neighbourhood design, natural and sustainable environment, transport and healthier food). It includes a series of five innovative diagrams, one for each of the five topic areas explored, to illustrate the associations between planning and design principles and health outcomes in order to assist discussions between public health and planning professionals.

Few studies have examined the challenges associated with integrating health evidence into spatial planning at local levels in England.^4^^,^^15^ The Design Council examined the barriers identified by built environment professionals in creating healthy places via a mixed-methods study.^4^ The authors reported that although built environment professionals had a good awareness of the importance of integrating health evidence into a design, several structural barriers including lack of capacity and resource at local levels and lack of synergy with public health teams often made it difficult to incorporate such design principles into the final built design solutions achieved on the ground.^4^ Others have reported that structural, political and economic factors still pose considerable obstacles to delivering healthy places at local level.^16^

Another study combined data from existing literature with data from workshops targeted at the wider built environment and health workforce, including interviews with developers, to understand ways of encouraging collaborations necessary for creating healthy places across public and private sectors.^17^ The authors reported that a lack of shared vision among the delivery agencies and a lack of staff capacity at local authority levels were key barriers to effective collaboration needed to deliver healthy places.^17^

This study was commissioned by PHE to:

- Examine the level of awareness of the Spatial Planning for Health resource among planners and public health teams at local authority levels in England.
- Explore the views of public health professionals and their planning colleagues on the barriers, facilitators and solutions needed to see improvements in integrating health evidence into planning at local levels.

## Methods

### Study design

A sequential exploratory mixed-methods study was conducted with the initial collection and analysis of qualitative data through in-depth semi-structured interviews, followed by the collection and analysis of quantitative survey data.^18^ Ethical approval for this research was granted by the UWE Bristol Ethics Committee (Project reference: HAS.18.10.044). This paper will mainly focus on qualitative findings.

### Participant recruitment: qualitative phase

To encourage representation and feedback from teams working across England, a purposive sample of public health and planning teams from each of the nine PHE Centres was selected using existing networks and links. A series of in-depth semi-structured ‘joint’ two-person interviews were conducted with a public health professional with portfolio responsibilities for health and planning and a planning professional with experience of working with public health colleagues in local authority settings. The joint interview approach was utilized to explore the nature of existing collaborations and investigate the challenges faced within and between disciplines. This interview approach is useful for generating more comprehensive data and eliciting shared or different understanding.^19^

In addition to the joint interviews described above, in-depth semi-structured interviews were conducted with public health professionals specializing in each of the five built and natural environment topics areas identified in Spatial Planning for Health (neighbourhood design, housing, healthier food, natural and sustainable environment and transport) to elicit their views on coverage in their areas of expertise. Individuals were purposively selected using existing networks and links but were predominantly drawn from PHE leads in these areas.

Potential interviewees were invited via email to participate in a face-to-face or telephone interview. An overview of participants is presented in Table 1.

**Table 1:** Phase 1 participant recruitment for qualitative interviews (*n* = 19)

Regions in England	Interview format
London	One-to-one
South-east	Joint interview
South-west	Joint interview
North-west	Joint interview
North-east	No interview conducted
West Midlands	Joint interview
East Midlands	Joint interview
Yorkshire and the Humber	One-to-one
East of England	Joint interview
Topic area specialists	
Neighbourhood design	One-to-one
Housing	One-to-one
Healthier food	One-to-one
Natural and sustainable environment	One-to-one
Transport	One-to-one

### Data collection and analysis of qualitative data

A semi-structured interview guide was developed for both participant groups to explore the challenges of integrating health evidence into planning at a local level. The interview guide was piloted with a public health professional and a planning colleague, both working in a local authority setting. Interviews were conducted between November 2018 and February 2019 and lasted between 30 minutes and 60 minutes. All interviews were audio-recorded, transcribed and imported into NVivo 12. The data from both groups were analysed together using thematic analysis.^18^ Findings from the interviews were used to develop an online survey for a larger pool of local public health and built environment professionals. Findings from the interviews were triangulated with themes emerging from round-table discussions at PHE’s first Spatial Planning for Health Seminar, held in March 2019.

### Participant recruitment: survey with public health and planning professionals

An online survey was created in Qualtrics Survey Software and was live for 3 weeks in April 2019. Potential participants were identified by PHE and the research team and contacted via existing mailing lists held by PHE and the research team, respectively, for data protection, and a link to the survey was shared on Twitter. Survey questions were derived from salient themes identified through the analysis of interview data and sought to explore:

a) Participants’ level of awareness and use of the *Spatial planning for Health* resource as well as other spatial planning for health tools
b) Barriers and facilitators associated with the implementation of health and spatial planning evidence at a local level
c) Recommendations for improving future implementation of health and spatial planning evidence at a local level

### Data collection and analysis of quantitative data

The survey was piloted with two public health professionals working in a local authority setting before the final version was made available. Survey data were extracted and imported into IBM SPSS Statistics v 22.0 for descriptive analysis.

## Results

Six semi-structured joint interviews were conducted with 12 public health and planning professionals working in local authorities. Due to time and resource constraints, 2 one-to-one interviews were conducted with public health professionals from London and Yorkshire and the Humber regions. Five interviews were conducted with specialists in each of the five topic areas covered by the Spatial Planning for Health resource.

The online survey yielded 162 responses from public health and built environment professionals. Due to the nature of the online survey, there is no denominator, and it is difficult to establish a response rate. Nearly half of the participants were public health professionals (*N* = 77, 48%) while 16% were planning policy planners (*N* = 27). Further details on participants for both research phases can be found in Tables 1 and 2.

**Table 2:** Phase 2: characteristics of participants in the online survey (*n* = 162)

Respondent characteristics	Number of respondents	Percentage
Role		
Public health professional	77	48
Planning policy planner	27	17
Other	25	15
Development management	6	4
Transport planning professional	6	4
Housing	6	4
Private sector consultant	4	3
Director of public health	3	2
Architect	2	1
Planner in government department	2	1
Urban designer	2	1
Main area of responsibility		
South-west	45	28
National	20	12
South-east	14	9
London	11	7
North-east	6	4
North-west	5	3

**Fig. 1:**
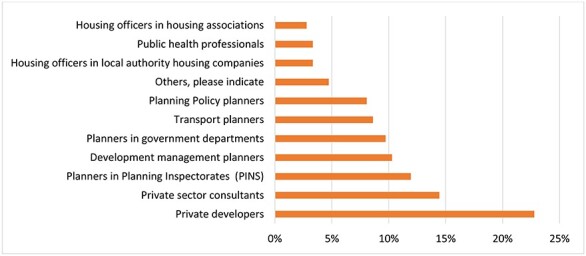
Organizations/professionals perceived to impede spatial planning and health integration (*n* = 162).

### Awareness of existing spatial planning and health guidance

The majority of respondents (*N* = 102, 63%) indicated that they were aware of the *Spatial Planning for Health* resource, while 37% of respondents were unaware (*N* = 60). The analysis revealed that 72% of public health professionals had heard of the resource compared with 56% of planning and built environment professionals. Findings from the survey corroborated interview findings, where nearly all public health professionals were aware of the resource but fewer planning professionals knew of its existence.

Respondents were asked to indicate their awareness of other existing spatial planning and health guidance from a list of relevant organizations. Guidance published by PHE recorded the highest number of responses (*N* = 70, 43%) followed by guidance from the Town and Country Planning Association (TCPA) (*N* = 61, 38%), Royal Town Planning Institute (RTPI) (*N* = 49, 30%), National Institute for Health and Care Excellence (NICE) (*n* = 44, 27%) and London HUDU (*N* = 41, 25%).

### Barriers associated with the implementation of health and spatial planning evidence base at a local level

The top three organizations/professionals perceived by survey respondents to impede the integration of spatial planning and health evidence at the local level were private developers, private sector consultants and planners in Planning Inspectorates (PINS) (Fig. 1).

Findings from the interviews provided insight into barriers to integrating health evidence into planning at local levels. Five areas were identified as critical. Firstly, there was a difference between public health and planning professions in their understanding and the use of evidence that was highlighted as a key barrier to collaborative working between public health and planning professionals. Planning professionals emphasized that policy and national standards are the most important sources of evidence, whilst public health professionals cited research evidence as most important.

‘With public health, evidence is king, and with planning, policy is king’. (Public health professional)

Secondly, economic arguments with developers were seen as a key barrier, with practitioners noting that developers would consider the statutory obligations but are less concerned with intangibles such as health that can impact on their profit margin.

‘It’s really hard to get a developer to think of valuation in anything but a monetary value’. (Planning professional)

Thirdly, some practitioners expressed concern that a lack of political support at the local level makes it difficult to influence local policies that ensure health is appropriately integrated into spatial planning.

‘I think some of the outcomes haven’t been as positive as we’d like because people aren’t prepared to make those difficult decisions because they’re worried about losing their seat’. (Public health professional)

Practitioners also argued that existing legislation is not strong enough to see substantial improvements in healthy place-making and that stronger legislation with explicit links to health integration is needed to engage with developers.

It’s all well and good having a document, but if we have got no means of it having traction with discussions with a developer, they’ll just say, ‘Thank you, but no’. (Planning professional)

Finally, issues of resource and capacity at local authority level were identified, with concerns raised about the impacts of reduced local authority budgets on the availability of resources and on the skillset needed to support collaborative work between public health and planning.

Survey participants were asked to indicate the perceived importance of barriers identified from the interview phase. Nine out of 10 respondents agreed that a lack of evidence that can be translated to practice at the local level is an important barrier to health integration into spatial planning at the local level; 89% of respondents considered the reduced capacity to be a major barrier (Table 3).

**Table 3:** Barriers in implementing research on healthy planning into practice at the local level (*n* = 162)

Barriers	Important (% of responders)	Neither important nor unimportant (% of responders)	Unimportant (% of responders)
Existing evidence is not translatable to practice at the local level	91%	19%	3%
Lack of resource and capacity at the local authority level	89%	6%	5%
Quality versus quantity: prioritizing the number of houses over the impact on health	89%	6%	5%
Communication and cultural gap between planners and public health professionals	85%	19%	5%
Lack of monitoring and evaluation of planning decisions	81%	15%	5%
Disconnect between government agencies responsible for providing leadership on spatial planning and health	79%	20%	6%
Lack of a designated funding stream for green infrastructure	78%	14%	2%
Political priorities and buy-in from local politicians	78%	9%	2%
Lack of robust planning guidance or regulation	72%	6%	6%
Lack of partnership structure required to deliver healthy places	71%	22%	9%
Lack of understanding/engagement with local public health priorities and needs	70%	20%	11%
Evidence exists but very often planners and stakeholders aren’t aware	70%	20%	11%
Planning inspectors not supporting decisions	67%	20%	13%

### Facilitators associated with the implementation of health and spatial planning evidence base at a local level

**Fig. 2:**
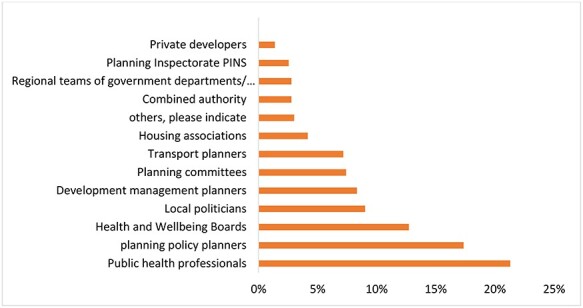
Organizations/professionals perceived to facilitate spatial planning and health integration (*n* = 162).

The top three organizations/professionals perceived by survey respondents to facilitate spatial planning and health integration at the local level were public health professionals, planning policy planners and health and well-being boards (Fig. 2).

Insight into facilitators of integrating health evidence into planning at the local level was obtained from interviews. Four areas were identified as critical. First, building relationships with developers was seen as important to promote values of healthy place-making.

‘The other group that we really need to engage with are the developers, the designers of the buildings, the commercial sector organizations that design and build the developments’. (Topic area specialist)

Secondly, there was seen to be a need in articulating the wider benefits for multiple stakeholders: practitioners identified that an important step to addressing siloed working across various sectors is to articulate the wider benefit of integrating health into planning to multiple stakeholders including developers, local authority, the NHS and other sectors.

‘I think what we need to get better at articulating in the research, is how actions that are being proposed will have multiple outcomes so they will be attractive to developers, they will increase environmental sustainability, they will increase the attainment of good health, healthy lifestyles and health outcomes’. (Topic area specialist)

Thirdly, both public health and planning professionals agreed that simplifying the presentation of evidence in terms of the language and accessibility to both fields enables collaborative working. Finally, there was seen to be a need to integrate health into the design of the Local Plan at an early stage, so it was not considered as an afterthought.

Survey participants were asked to indicate how important they perceived the opportunities identified from the interview phase. Nearly all respondents (96%) agreed that integrating health into the Local Plan is an important facilitator of healthy spatial planning; Table 4 shows respondents’ assessment of some potential facilitators and their level of importance.

**Table 4:** Potential facilitators in implementing research on healthy planning into practice at the local level (*n* = 162)

Potential facilitators	Important (%)	Neither important nor unimportant (%)	Not important (%)
Integrating health into the local plan	96%	3%	1%
Shared vision of delivery by those involved in spatial planning decisions	95%	4%	1%
Simplifying the evidence on planning and health to aid communication between public health and planners	86%	9%	4%
Building relationships with developers to improve health awareness	84%	10%	6%
Community engagement through consultations with local communities	82%	9%	8%
Developing good partnership with developers/private sector that takes a long-term view	81%	13%	5%
Forward funding of transport infrastructures	79%	15%	6%
Engaging housing association in place-making and health	74%	17%	8%
Improved synergy between public health and resilience planning	73%	16%	11%
Joined up collaborations with multiple stakeholders including academics	69%	23%	8%
Incentivizing developers	68%	24%	8%
Streamlining the process for developers through the use of checklists	63%	26%	11%

### Recommendations for improving future implementation of health and spatial planning evidence at a local level

Survey respondents were asked to rank a list of recommendations identified during the interview stage for the future development and implementation of health and spatial planning evidence. Improving national guidance and having stronger policies for place-making and health were ranked as the most important recommendations, while organizing networking events was ranked as the least important recommendation (Table 5).

**Table 5:** Rank of future recommendations for integrating health evidence into planning at local levels (from highest to lowest)

Rank	Future recommendations
1	Improved national guidance and stronger policies for place-making and health
2	Engaging politicians with healthy spatial planning
3	Taking a holistic view of health and place
4	Articulating the wider benefits to multiple stakeholders
5	Strategic partnerships between public health and planning agencies at national level
6	Funding high-quality research with practical application at the local level
7	Research on cost-benefit of healthy places for various sectors
8	Creating a central repository of good practice
9	Joint Continuing Professional Development (CPD) events/training for public health and built environment professionals
10	Recruiting strong champions and advocates for spatial planning and health
11	Organizing networking and knowledge exchange events

## Discussion

### Main findings of this study

This study explored barriers and facilitators to integrating health evidence into spatial planning at local authority levels and examined the awareness and use of the PHE Spatial Planning for Health resource. The findings from this study demonstrated high awareness and use of the Spatial Planning for Health resource across many different professionals across a wide range of local authorities, albeit with a greater reach within the public health compared with the built environment professions. There may be scope for more collaborative working with other built environment organizations to further extend its reach. This formal review of the use of the resource is one way of assessing their impact on getting research into practice and supporting how future publications might be framed.

The findings from this study also demonstrated that difficulties in translating evidence, for different audiences with differing needs, were a key barrier in getting evidence into practice at the local level. A lack of resource and skillset to support collaborative work between public health and planning was reported as the second most important barrier facing local professionals. This finding aligns with findings from other research by the Design Council and the TCPA. ^4^^,^^15^ Findings from this research validate the UCL Lancet Commission’s^20^ perspective that decision-makers in planning healthy cities are not in direct control but are participants in a system responding and managing the outcomes and effects of interventions as they occur.

Findings from this research highlight some essential actions for consideration by specific stakeholders. There is a need to ensure that spatial planning and health resources meet the practical needs of both planning and public health professionals. Only a quarter of respondents reported awareness of guidance from NICE despite its well-recognized status. Findings from this research suggest that whilst planners require more concise and visual information, public health professionals rely on robust and detailed analysis of evidence. National and local bodies should recognize these different needs when developing future resources and the impact they will have on document format, length and style.

There is a need to integrate local health and well-being needs and priorities into the Local Plan and decision-making process. Heads of Planning play an essential role in ensuring that Local Plans are up to date and meet not only the generic health and well-being requirements in the NPPF and the National Planning Practice Guidance^8^ but also link to local needs as outlined in Joint Strategic Needs Assessments (JSNAs). This also requires that Directors of Public Health and their teams should ensure that all health and well-being strategies (and healthcare strategies) refer to the environmental aspects of disease causation and how the built environment can be modified to support health and well-being. This aligns with recommendations from TCPA on the importance of referring to specific health needs identified in local JSNAs and health and well-being strategies in the development of Local Plans.^17^

Developing a common shared understanding of different professional perspectives is crucial for maximizing the effectiveness of decision-making and aiding joint working within local authorities. A joint basic understanding of the impacts of the built environment on health and the systems and processes which are used by both built environment and public health professionals through a local training programme is pivotal in addressing cultural gaps.^4^^,^^17^ Training could be jointly delivered with key partners such as the professional institutes and universities and targeted across the spectrum of the career path from undergraduate modules to professional continuing professional development.

Political support is essential to ensure that improvements to health underpin all planning decisions at the local level. Political support from elected members and clear corporate priorities were identified as crucial determinants of the extent to which health is integrated into spatial planning. It is therefore important to engage with local politicians in discussions on healthy spatial planning. Planning and public health organizations should be aware of the need to prepare evidence and guidelines that are easily accessible to politicians as well as professionals. An example would be the ‘Tackling Obesity Through Planning and Development’ document published by the Local Government Association.^21^

There was a perceived need to improve access to existing wealth of knowledge with strong support for a central repository of good practice for sharing good practice across both disciplines. Practitioners would appreciate clearer signposting and access to this information, and there are suggestions that organizations or institutions with greater capacity such as universities can take on this role.

### What is already known on this topic?

There are barriers and opportunities in integrating health into spatial planning at local level. Barriers include those of communication and a cultural gap between public health professionals and planners.^4^^,^^5^^,^^11^ Carmichael *et al*. identified communications and cultural barrier, lack of funding and skills gap as barriers of health integration into urban spatial planning via impact assessment.^22^ The need for a central repository for sharing good practice and locating evidence that can be applied locally was also reported in the study by Design Council.^4^

### What this study adds?

This study evaluated the use of a targeted resource aimed at addressing some of the existing barriers to integrating health into planning. The joint interview approach adopted by this current study provided in-depth insight into awareness and use of this resource as well as exploring the barriers and opportunities to effectively use health and spatial planning evidence by public health and planning teams in local authority settings. Whilst evidence is essential, it is clear that a whole system approach is required to address the barriers identified. Improved legislation and policies with clear and explicit links to health are necessary to empower built environment professionals with the leverage needed to secure health integration with developers.

### Limitations of this study

There were some limitations in our research design. Due to time and resource constraints, we were unable to conduct joint interviews in two regions. It was also not possible to interview a public health or planning professionals in the North-east region, despite attempts by the research team to do so. This might imply limitations in the robustness of the interview findings; however, as the findings from all the interviews conducted were consistent, it is unlikely that we would have missed any contradictory findings.

## Conclusion

Findings from this research suggest that the Spatial Planning for Health resource is well recognized by both public health and planning teams. It has proved to be a useful resource for local practitioners and demonstrates how universities can work closely with government organizations to produce robust, detailed evidence reviews which provide the basis for ‘translation’ into more user-friendly documents for a more local, professional audience. However, to implement the evidence contained in this resource—and indeed other existing spatial planning and health resources—at local level effectively, further work is required to address the structural and political barriers identified in this research.

## Notes

**Janet Ige-Elegbede,** Research Fellow in Public Health

**Emma L. Bird,** Senior Lecturer in Public Health

**Selena Gray,** Professor of Public Health

**Jennifer S. Mindell,** Professor of Public Health

**Paul Pilkington,** Associate Professor in Public Health

**Michael Chang,** Programme Manager—Planning and Health

**Aimee Stimpson,** National Lead, Healthy Places

**Dominic Gallagher,** Programme Support Officer—Healthy Places team

**Carl Petrokofsky,** Public Health Specialist Advisor

## References

[1] Bird EL, Ige JO, Pilkington P et al. Built and natural environment planning principles for promoting health: an umbrella review. BMC Public Health 2018;18:930. 30055594 10.1186/s12889-018-5870-2 PMC6064105

[2] Ige J, Pilkington P, Orme J et al. The relationship between buildings and health: a systematic review. J Public Health 2019;41:e121–32. 10.1093/pubmed/fdy138 PMC6645246 30137569

[3] Grant M, Braubach M. Evidence review on the spatial determinants of health in urban settings. In: Urban Planning, Environment and Health: From Evidence to Policy Action, Meeting Report. WHO Regional Office for Europe, 2010, pp. 22–97.

[4] Design Council . Healthy Placemaking Report*.* 2018. www.designcouncil.org.uk/resources/report/healthy-placemaking-report (12 August 2019, date last accessed).

[5] Pilkington P, Grant M, Orme J. Promoting integration of the health and built environment agendas through a workforce development initiative. Public Health 2008;122:545–51. 18462767 10.1016/j.puhe.2008.03.004

[6] Barton H, Grant M. Urban planning for healthy cities. J Urban Health 2013;90:129–41. 22714703 10.1007/s11524-011-9649-3 PMC3764272

[7] Carmichael L, Townshend TG, Fischer TB et al. Urban planning as an enabler of urban health: challenges and good practice in England following the 2012 planning and public health reforms. Land Use Policy 2019;84:154–62.

[8] Ministry of Housing , Communities and Local Government. London: National Planning Policy Framework, 2019. https://assets.publishing.service.gov.uk/government/uploads/system/uploads/attachment_data/file/810197/NPPF_Feb_2019_revised.pdf (27 October 2019, date last accessed).

[9] Chang M, Ellis H, Mannion F. Spatial Planning for Health: A Guide to Embedding the Joint Strategic Needs Assessment in Spatial Planning. 2010. www.tcpa.org.uk/data/files/spatial_planning_for_health.pdf (1 September 2019, date last accessed).

[10] Camden Planning for Health and Wellbeing . 2017. https://www.camden.gov.uk/documents/20142/4834411/Appendix+11+Planning+for+Health+and+Wellbeing.pdf/cf4a3362-194b-270e-3144-a36bb7be9a07 (7 September 2019, date last accessed).

[11] Lake AA, Henderson EJ, Townshend TG. Exploring planners and public health practitioners views on addressing obesity: lessons from local government in England. Cities Health 2017;1:185–93.

[12] Public Health England . Spatial Planning for Health: Evidence Review. 2017. https://assets.publishing.service.gov.uk/government/uploads/system/uploads/attachment_data/file/729727/spatial_planning_for_health.pdf (1 September 2019, date last accessed).

[13] NHS London Healthy Urban Development Unit . HUDU Planning for Health: Rapid Health Impact Assessment Tool. 2013. https://www.healthyurbandevelopment.nhs.uk/wp-content/uploads/2013/12/HUDU-Rapid-HIA-Tool-Jan-2013-Final.pdf (7 September 2019, date last accessed).

[14] NHS England . Putting Health into Place. 2019. https://www.england.nhs.uk/publication/putting-health-into-place-executive-summary/ (24 October 2019, date last accessed).

[15] TCPA . Developers and Wellbeing Securing constructive collaboration and consensus for planning healthy developments. A Report from the Developers and Wellbeing Project 2018. https://www.tcpa.org.uk/Handlers/Download.ashx?IDMF=b9a54964-9cf5-49d4-8ef4-095d2436719f (1 September 2019, date last accessed).

[16] Le Gouais A, Foley L, Ogilvie D et al. Decision-making for active living infrastructure in new communities: a qualitative study in England. J Public Health https://academic.oup.com/jpubhealth/advance-article/doi/10.1093/pubmed/fdz105/5573986 (30 September 2019, date last accessed). 10.1093/pubmed/fdz105 PMC7435215 31565741

[17] TCPA . The State of the Union: Reuniting Health with Planning in Promoting Healthy Communities. 2019. www.tcpa.org.uk/Handlers/Download.ashx?IDMF=cb4a5270-475e-42d3-bc72-d912563d4084 (12 August 2019, date last accessed).

[18] Creswell JW, Plano Clark VL. Designing and Conducting Mixed Methods Research. London: Sage, 2018.

[19] Arksey H . Collecting data through joint interviews. Social Research Update No. 15 1996.

[20] Rydin Y . Shaping cities for health: complexity and the planning of urban environments in the 21st century. The Lancet 2012;379.9831:2079–108. 10.1016/S0140-6736(12)60435-8 PMC3428861 22651973

[21] Chang M. Building the Foundations: Tackling Obesity Through Planning and Development, 3rd edn. London: Local Government Association, 2016. https://www.local.gov.uk/sites/default/files/documents/building-foundations-tack-f8d.pdf (24 October 2019, date last accessed).

[22] Carmichael L, Barton H, Gray S et al. Integration of health into urban spatial planning through impact assessment: identifying governance and policy barriers and facilitators. Environ Impact Assess Rev 2012;32:187–94.

