# Erratum to: Exploring the views of planners and public health practitioners on integrating health evidence into spatial planning in England: a mixed-methods study

**DOI:** 10.1093/pubmed/fdab379

**Published:** 2021-10-19

**Authors:** Janet Ige-Elegbede, Paul Pilkington, Emma L Bird, Selena Gray, Jennifer S Mindell, Michael Chang, Aimee Stimpson, Dominic Gallagher, Carl Petrokofsky

**Affiliations:** Centre for Public Health and Wellbeing, The University of the West of England, Stoke Gifford BS16 1QY, UK; Centre for Public Health and Wellbeing, The University of the West of England, Stoke Gifford BS16 1QY, UK; Centre for Public Health and Wellbeing, The University of the West of England, Stoke Gifford BS16 1QY, UK; Centre for Public Health and Wellbeing, The University of the West of England, Stoke Gifford BS16 1QY, UK; Department of Epidemiology and Public Health, UCL, London WC1E 6BT, UK; Healthy Places, Priorities and Programmes Division, Health Improvement Directorate, Public Health England, London SE1 8UG, UK; Healthy Places, Priorities and Programmes Division, Health Improvement Directorate, Public Health England, London SE1 8UG, UK; Healthy Places, Priorities and Programmes Division, Health Improvement Directorate, Public Health England, London SE1 8UG, UK; Healthy Places, Priorities and Programmes Division, Health Improvement Directorate, Public Health England, London SE1 8UG, UK

In the originally published version of this manuscript, there was an error in the surname of the corresponding author, Dr Janet Ige-Elegbede. In the HTML version, `Ige' erroneously started with a lower case `L' instead of a capital `I'.

The Publisher apologizes for this error. It has now been corrected.

